# Hyaluronan Synthases’ Expression and Activity Are Induced by Fluid Shear Stress in Bone Marrow-Derived Mesenchymal Stem Cells

**DOI:** 10.3390/ijms22063123

**Published:** 2021-03-18

**Authors:** Sebastian Reiprich, Elif Akova, Attila Aszódi, Veronika Schönitzer

**Affiliations:** Experimental Surgery and Regenerative Medicine (ExperiMed), Department of General, Trauma and Reconstructive Surgery, Munich University Hospital, Ludwig-Maximilians-University, 80336 Munich, Germany; Sebastian.Reiprich@med.uni-muenchen.de (S.R.); Elif.Akova@med.uni-muenchen.de (E.A.); Attila.Aszodi@med.uni-muenchen.de (A.A.)

**Keywords:** mesenchymal stem cells, mechanotransduction, osteogenic differentiation, biomineralization, hyaluronan synthases

## Abstract

During biomineralization, the cells generating the biominerals must be able to sense the external physical stimuli exerted by the growing mineralized tissue and change their intracellular protein composition according to these stimuli. In molluscan shell, the myosin-chitin synthases have been suggested to be the link for this communication between cells and the biomaterial. Hyaluronan synthases (HAS) belong to the same enzyme family as chitin synthases. Their product hyaluronan (HA) occurs in the bone and is supposed to have a regulatory function during bone regeneration. We hypothesize that HASes’ expression and activity are controlled by fluid-induced mechanotransduction as it is known for molluscan chitin synthases. In this study, bone marrow-derived human mesenchymal stem cells (hMSCs) were exposed to fluid shear stress of 10 Pa. The RNA transcriptome was analyzed by RNA sequencing (RNAseq). HA concentrations in the supernatants were measured by ELISA. The cellular structure of hMSCs and HAS2-overexpressing hMSCs was investigated after treatment with shear stress using confocal microscopy. Fluid shear stress upregulated the expression of genes that encode proteins belonging to the HA biosynthesis and bone mineralization pathways. The HAS activity appeared to be induced. Knowledge about the regulation mechanism governing HAS expression, trafficking, enzymatic activation and quality of the HA product in hMSCs is essential to understand the biological role of HA in the bone microenvironment.

## 1. Introduction

Biomineralization is defined as a process by which living organisms control the formation of hierarchically structured minerals. In animals, the ability to produce biominerals evolved around 550 million years ago [1]. Interestingly, biomineralization arose independently multiple times in various phyla through the attachment of amorphous precursor nanoparticles [2,3], implicating that the chemical, physical and molecular mechanisms responsible for mineralization are developed by convergent evolution. The extra- and intracellular processes must be fine-tuned inside specialized cells generating mineral deposits such as epithelial mantle cells in molluscs or osteoblasts in the bone.

During the evolution of biomineralization, an intricate set of mechanisms which convert mechanical stimuli into biochemical cascades, termed mechanotransduction, has been developed as a general machinery for the regulation of biomaterial designs [4]. The phenomenon of mechanotransduction is an integral part of the general process of skeletal adaptation to mechanical loads as described by Wolff’s law [5]. Bone is a dynamic tissue and its mineral density, along with chemical signals, changes in response to the applied forces on the bone. Mechanically induced bone remodeling, the physiological balance between bone formation and bone resorption, is primarily attributed to osteocytes and osteoblasts; however, other cells in the bone marrow such as multipotent mesenchymal stem cells (MSCs) are also mechanically sensitive [6]. It has been shown that various types of fluid shear stress (steady, pulsatile, oscillatory) facilitate MSC osteogenic differentiation in two-dimensional (2D) in vitro culture systems in a range of 0.1–2 Pa [7]. In vivo, numerical poroelastic modeling predicted that mechanical loading-induced interstitial fluid movement through the lacunar-canalicular network of the bone osteoid generates a fluid shear stress between 0.8 and 3 Pa [8,9]. However, volumetric- and time-averaged shear stress calculations upon cyclic loading in porcine trabecular bone marrow, where MSCs reside, ranged from 2.14 to 15.57 Pa [10].

Mechanotransduction also plays a regulatory role in the formation of molluscan shell. In general, an organic matrix is necessary for the nucleating minerals and crystals. Chitin, a homo-polymer of β- (1-4) linked N-acetylglucosamine molecules, is the main part of this matrix and fulfills an important function in the formation and functionality of mollusc larval shells [11,12]. The molluscan myosin-chitin synthases, which are responsible for the production of chitin, are unique transmembrane enzymes with an N-terminal myosin motor domain and highly conserved regions of charged amino acids. The complexity of the enzymatic structure indicates that the myosin-chitin synthases coordinate the intracellular processes of the shell-forming epithelial cells and the extracellular mineralization events via mechanical signal transduction [4,13,14].

Hyaluronan (HA), just like chitin, is a linear polysaccharide, which consists of alternating residues of β-D-(1-4)-N-acetylglucosamine and β-D-(1-3)-glucuronic acid. HA plays important roles in skeletal biology, influencing processes such as migration and condensation of skeletogenic progenitor cells, limb development, joint cavity formation and longitudinal bone growth [15,16]. HA is synthesized by adult human MSCs (hMSCs) [17,18], osteoclast-like cells [19,20], osteoblasts [21] and osteocyte-like cells [22,23] and is a main component of the bone marrow. However, its precise function in the bone remodeling process is not yet known [24]. Furthermore, HA may act as a regulator of mineralization, although it does not change the rate of crystal growth [25]. HA binds hydroxyapatite in calcified cartilage and bone. HA of high molecular weight (>500—~2000 kDa) stimulates proliferation and mineralization in rat osteoblasts, while HA of low molecular weight (<8 kDa) increases only proliferation [26]. Due to its abundant negative charges, HA might be able to bind calcium in high concentration to induce crystallization. HA could also have a function in mineralization initiation by space capture and/or matrix organization [27,28]. HA is produced by hyaluronan synthases (HAS) which belong to the glycosyltransferases 2-family together with chitin synthases and cellulose synthases. The three known mammalian HAS isoforms (HAS1, HAS2 and HAS3) are plasma membrane glycosyltransferases sharing 55–70% structural identity. The isoenzymes have seven membrane-spanning domains and a large cytoplasmic region with the active enzyme and substrate binding sites. HAS isoforms differ in the molecular size of their product: HAS1 and HAS2 polymerize HA chains of around 2 × 10^6^ Da, whereas HAS3 produces shorter chains of 1 × 10^5^ Da to 1 × 10^6^ Da. Human bone marrow-derived MSCs express all three HAS isoforms and the HA receptor CD44.

The question arises as to whether HA plays a similar regulatory role in bone formation to chitin in molluscan shell formation. However, the processes in the bone might be more complicated, since bone remodels in a way unlike molluscan shell. In this study, we applied fluid shear stress to bone marrow-derived hMSCs as mechanical stimuli and analyzed the complete set of RNA transcripts and the activity of HASes in hMSCs. In some bone diseases, the HA content in the bone or HAS expression in hMSCs is changed [18,29,30]. Understanding the regulatory mechanisms of HAS in hMSCs will provide therapeutic starting points for an improved fracture healing in patients suffering from one of these bone diseases.

## 2. Results

### 2.1. Cell Morphology of hMSCs after Exposure to Fluid Shear Stress

**Figure 1:**
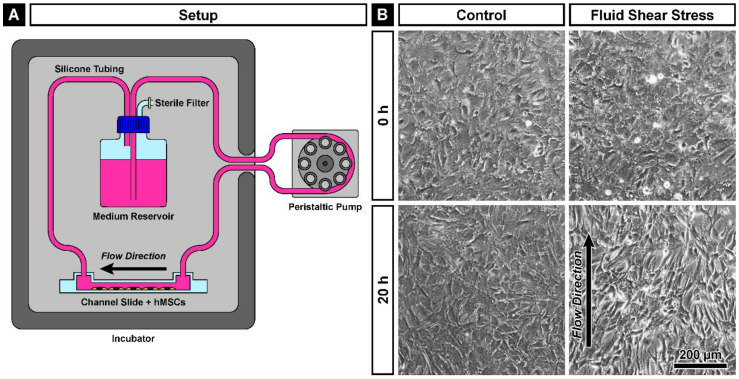
Exposure of human mesenchymal stem cells (hMSCs) to fluid shear stress. (**A**) Schematic drawing of the experimental setup. All components besides the peristaltic pump were kept in a humidified incubator at 37 °C and 5% CO_2_. (**B**) Phase-contrast microscopy of hMSC-2. After 20 h of shear stress, an arrangement of the cells along the flow direction (arrow) is visible. Note the spindle-like shape of the cells after the shear stress.

To induce mechanotransduction via fluid shear stress in hMSCs obtained from the bone marrow of four healthy donors (hMSC-2, hMSC-15, hMSC-19 and hMSC-20), cells were cultured on fibronectin-coated channel slides which were connected to a medium-containing tubing system driven by a peristaltic pump. As a control, hMSCs were cultured on fibronectin-coated 24-well plates under static conditions. The whole system, except the pump itself, as well as the control cells were kept in a humidified incubator at 37 °C and 5% CO_2_ (Figure 1A). Cells after seeding were allowed to adhere for 6.5 h before shear stress of 10 Pa (100 dyn/cm^2^) was applied for a duration of 20 h. This pressure level matches the range of volumetric mean marrow shear stress in porcine femurs of 7.1 ± 6.2 Pa during stress relaxation and 9.6 ± 6.9 Pa during cyclic loading as demonstrated by Metzger et al. [10]. Differences in shape and alignment between control and shear stress-exposed hMSCs were clearly visible under phase-contrast microscopy. In comparison to the cuboidal shape of the static cells, the hMSCs upon shear stress had a longer, more spindle-like morphology and aligned along the flow direction (Figure 1B). There was no difference between hMSCs obtained from different donors.

### 2.2. Transcriptional Changes in hMSCs Exposed to Shear Stress

**Figure 2:**
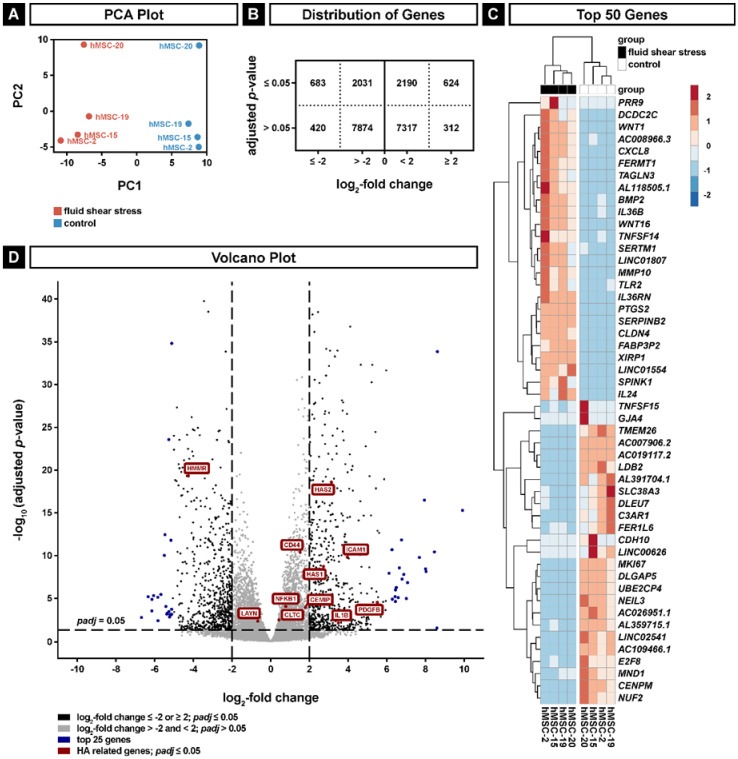
Next-generation RNA sequencing analyses of hMSCs exposed to fluid shear stress. (**A**) Principal component analysis (PCA) for fluid shear stress (PC1) and donors (PC2). (**B**) Distribution of genes with more than ten reads; genes are grouped according to significance (adjusted *p* ≤ 0.05) and log_2_-fold change (≤−2 or ≥2). (**C**) Heatmap of the 50 most significantly regulated genes with the highest and lowest log_2_-fold changes. (**D**) Volcano plot of genes with more than ten reads. Dashed lines show thresholds for significance (*p* ≤ 0.05) and log_2_-fold change (≤−2 or ≥2). The top significantly regulated genes with the highest and lowest log_2_-fold change are shown in blue; the significantly changed genes of the hyaluronan biosynthetic pathway (GO:0030213) and the significantly changed hyaluronan (HA) receptors *CD44*, *HMMR*, *ICAM1* and *LAYN* are shown in red.

To assess the impact of shear stress on gene expression, we performed next-generation RNA sequencing of the hMSC transcriptome obtained from four different donors. Principal component analysis (PCA) showed a clear segmentation according to the applied fluid flow along PC1 (Figure 2A). Although the expression profiles of the donors along PC2 were different, these discrepancies remained comparable despite the application of fluid shear stress. For further analysis of the gene expression response to fluid flow, we investigated the significantly up- and downregulated genes in shear stress-exposed versus control hMSCs. The genes were filtered by a minimum of ten reads to exclude lower expression and the remaining mapped genes were considered as significantly changed with an adjusted *p*-value smaller than 0.05. As biological relevant changes in gene expression, we defined a log_2_-fold change smaller than -2 or higher than 2. This resulted in 683 significantly downregulated and 624 significantly upregulated genes (Figure 2B). A graphical presentation of the 25 most upregulated and the 25 most downregulated genes is depicted in the heat map (Figure 2C), while the volcano plot (Figure D) shows all genes with more than ten reads with cut-off lines for significance and biological relevance. In the volcano plot, the top 25 upregulated and top 25 downregulated genes are indicated in blue color. Among the prominent upregulated genes, we found *Wnt1*, *Wnt16* and *BMP2*, which are known to be involved in bone remodeling and to induce bone formation [31,32,33]; *TNFSF14*, which participates in the cellular response to a mechanical stimulus [34,35]; *PTGS2*, which is a regulator of bone metabolism [36] and upregulated upon shear stress in MSCs [37]; *CLDN4*, which is important for calcium-independent cell adhesion activity at tight junctions [38]; and *XIRP1*, which prevents actin depolymerization [39]. Distinguished downregulated genes were *LDB2*, which regulates cell migration [40]; *C3AR1*, which is involved in chemotaxis of mesenchymal stem cells [41]; *CDH10*, which encodes for a calcium-dependent cell adhesion protein [42]; and *MKI67*, *DLGAP5* and *NUF2*, which play roles in the cell cycle and cell proliferation [43,44,45].

In order to elucidate the function of HA in hMSCs treated with shear stress, we highlighted significantly changed genes of the hyaluronan biosynthetic pathway (GO:0030213) and the significantly changed HA receptors *CD44*, *HMMR*, *ICAM1* and *LAYN* with red color in the volcano plot (Figure 2D). The expression levels of *LAYN* and *HMMR* are decreased, indicating a potential inhibition of cell migration. The HA receptor layilin interacts with actin filaments and might promote cell migration; however, its exact biological function is not known yet [46]. The hyaluronan-mediated motility receptor, HMMR, is responsible for cell motility [47]. Interestingly, there is a clear tendency for upregulation of the hyaluronan synthase genes *HAS1* and *HAS2* and the hyaluronan receptors *ICAM1*, *IL1B* and *PDGFB* with a log_2_-fold change higher than 2. *ICAM-1* encodes for an Ig-like cell adhesion molecule, which mediates cell–cell adhesion or serves as a receptor for extracellular HA [48]. At the transcriptional level, the expression of hyaluronan synthases is regulated by numerous growth factors and cytokines, e.g., PDGF-BB [49] and IL1-β [50].

Altogether, in our experimental setup, hMSCs responded to fluid shear stress by increasing the expression of genes involved in bone formation, cell adhesion to the surface, cell–cell adhesion and hyaluronan biosynthesis. On the contrary, expression of genes associated with proliferation, the cell cycle and migration was diminished upon exposure to fluid flow.

### 2.3. Fluid Shear Stress Regulated Biological Pathways in hMSCs

**Figure 3:**
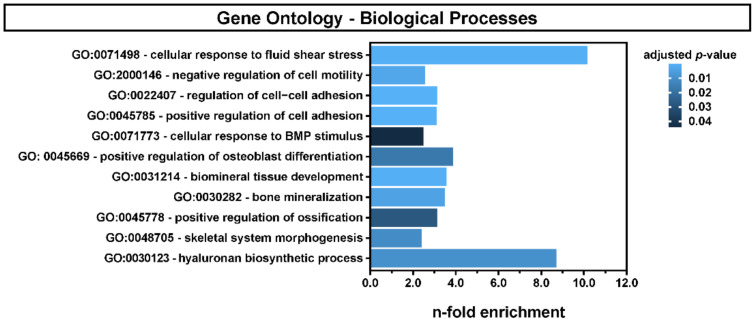
Gene set enrichment analysis of the 624 upregulated genes (adjusted *p* ≤ 0.05; log_2_-fold change ≥ 2). Overrepresented biological processes are involved in the morphogenesis of the skeletal system, biomineralization, HA biosynthesis and the response to fluid shear stress.

To further investigate the response of hMSCs upon mechanical stimulation with fluid shear stress, we performed Gene Ontology (GO) enrichment analysis of the 624 upregulated genes. Figure 3 shows a selection of differentially regulated biological processes involved in the positive regulation of the skeletal system development, biomineralization and HA biosynthesis. The enrichment score was calculated by dividing the sample frequency by the background frequency of genes annotated to a certain GO term. As a kind of control for a reaction to the mechanical stimulus, the GO term of “cellular response to fluid shear stress” was also significantly overrepresented.

**Figure 4:**
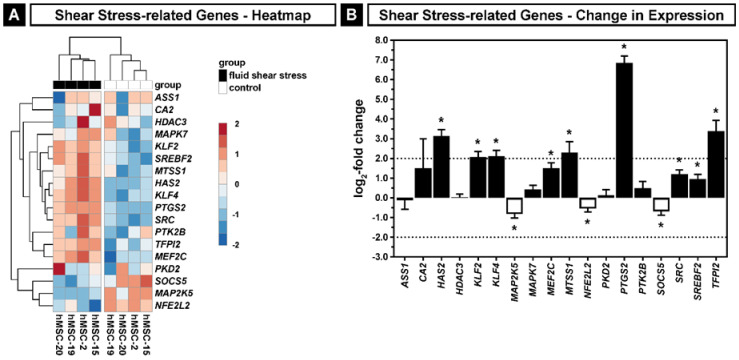
Cellular response to fluid shear stress (GO:0071498) in hMSCs. (**A**) Heatmap of the gene set. (**B**) Change in expression of the gene set. Black bars show upregulation of expression induced by fluid shear stress; white bars show downregulation; dashed lines indicate the thresholds for the log_2_-fold change of ≤−2 and ≥2; asterisks indicate an adjusted *p* ≤ 0.05. Error bars represent SE.

In detail, the genes which contribute to the “cellular response to fluid shear stress” (GO:0071498) showed a clear cluster of nine significantly upregulated genes (*HAS2*, *KLF2*, *KLF4*, *MEF2C*, *MTSS1*, *PTGS2*, *SRC*, *SREBF2*, *TFPI2*), six genes without changes (*ASS1*, *CA2*, *HDAC3*, *MAPK7*, *PKD2*, *PTK2B*) and three significantly downregulated genes (*MAP2K5*, *NFE2L2*, *SOCS5*) in hMSCs. Thus, we observed a clear tendency for a positive response to fluid shear stress. Although we found donor-dependent differences in the expression levels of the individual genes, all donors showed a reaction to the mechanical stimulus in the expression of the majority of genes with minor individual differences (Figure 4A). None of the downregulated genes had a log_2_-fold change (LFC) value smaller than −2, but *HAS2*, *KLF2*, *KLF4*, *MTSS1*, *PTGS2* and *TFPI2* exhibited a significant LFC value higher than 2 (Figure 4B).

In particular, the upregulation of *PTGS2* is a well-known marker for shear stress in hMSCs [37]. The transcription factor KLF4 regulates membranous and endochondral ossification during skeletal development [51]. *HAS2* belongs to the known genes which are induced by high shear stress, as it was demonstrated that the expression of HAS2 is increased by pulsatile, arterial-like shear stress, but not by lower shear stress in human umbilical vein endothelial cells [52].

**Figure 5:**
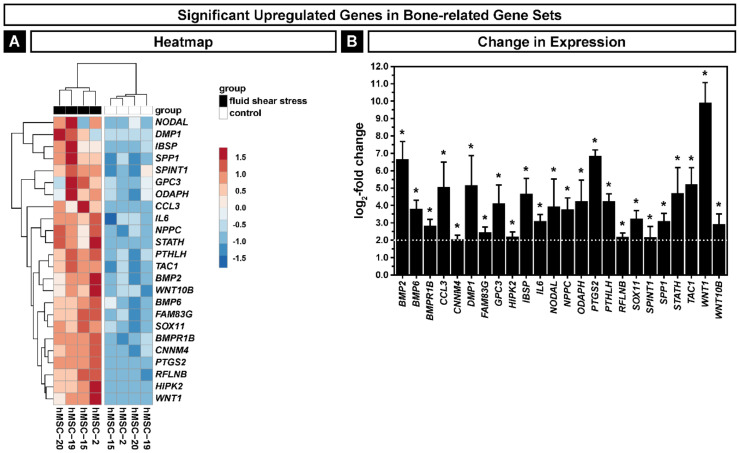
Significantly upregulated bone-related genes in hMSCs exposed to fluid shear stress. (**A**) Heatmap of the genes. (**B**) Change in expression of the genes. The dashed line indicates the threshold for the log_2_-fold change of ≥2; asterisks indicate an adjusted *p* ≤ 0.05. Error bars represent SE.

Mechanotransduction is known to be important for the regulation of osteogenic differentiation of MSCs [53]; therefore, we took a closer look at the bone-related genes contributing to the overrepresented pathways shown in Figure 3 (GO:0071773, GO:0045669, GO:0031214, GO:0030282 and GO:0045778). Our analysis revealed 24 significantly upregulated genes (Figure 5A,B) with a log_2_-fold change higher than 2. Bone morphogenetic protein (BMP) signaling is essential in bone formation and represented here by *BMP2*, *BMP6* and the receptor *BMPR1B*, which are involved in bone remodeling and osteoblast differentiation [33]. BMP2-induced osteogenesis is enhanced by the presence of IL6 in vitro and in vivo [54]. We also observed an upregulation of three genes (*DMP1*, *IBSP* and *SPP1*) coding for non-collagenous bone matrix proteins of the small integrin-binding ligand, the N-linked glycoprotein family [55], which are important for bone mineralization [56]. *WNT1* and *WNT10B* are also part of the significantly upregulated genes in these bone-related pathways, both involved in the generation and function of osteoblasts [31,57]. The gene set also contains the aforementioned *PTGS2* and *SOX11,* which are involved in early osteoblast differentiation [58]. We also detected an upregulation of *ODAPH*, which participates in the enamel mineralization of teeth [59], and *PTHLH*, a gene important for bone integrity [60]. The upregulation of these bone-related genes showed only a slight donor-to-donor variability (Figure 5A).

**Figure 6:**
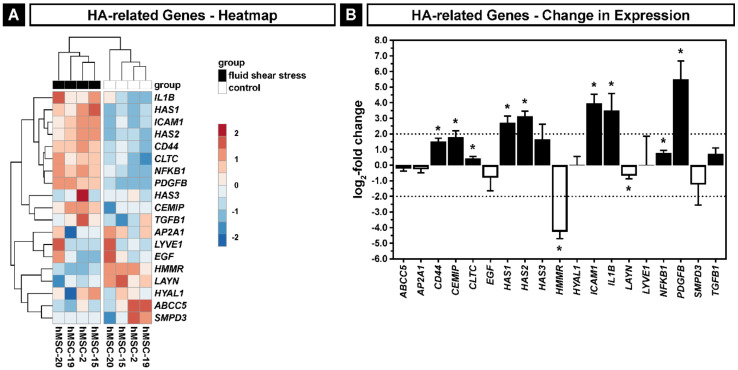
Hyaluronan biosynthetic process (GO:0030213) and five HA receptors (*CD44*, *HMMR*, *ICAM1*, *LAYN* and *LYVE1*) in hMSCs exposed to fluid shear stress. (**A**) Heatmap of the gene set. (**B**) Change in expression of the gene set. Black bars show an upregulation of expression by fluid shear stress; white bars show downregulation; dashed lines indicate the thresholds for the log_2_-fold change of ≤−2 and ≥2; asterisks indicate an adjusted *p* ≤ 0.05. Error bars represent SE.

To obtain a better understanding about the expression changes of the HA-related genes induced by the exposure to fluid shear stress, we selected the gene set of the hyaluronan biosynthetic process (GO:0030213) and the genes encoding for five HA receptors (*CD44*, *HMMR*, *ICAM1*, *LAYN* and *LYVE1*). Plotted as a heatmap, we found a group of nine significantly upregulated genes (*CD44*, *CEMIP*, *CLTC*, *HAS1*, *HAS2*, *ICAM1*, *IL1B*, *NFKB1*, *PDGFB*), seven genes with insignificant changes in expression (*ABCC5*, *AP2A1*, *EGF*, *HAS3*, *HYAL1*, *LYVE1*, *SMPD3*, *TGFB1*) and two genes showing a significant downregulation with an overall tendency to a positive LFC (Figure 6A). Whilst *HAS1*, *HAS2*, *ICAM1*, *IL1B* and *PDGFB* showed a significant LFC higher than 2, the expression of *HAS3* was not changed significantly. Only *HMMR* was significantly downregulated with an LFC smaller than −2 (Figure 6B). CD44, the best known HA receptor, is involved in several intracellular signaling pathways regulating cell–cell and cell–substrate adhesion, proliferation, motility, migration, nuclear signaling and HA endocytosis and degradation [61].

In summary, the bioinformatics analysis of the gene expression profiles of hMSCs upon shear stress demonstrates not only changes in the expression levels of the known gene set regulating the cellular response to fluid flow but also changes in the genes participating in bone development and HA synthesis. This might be an indication that the expression of HASes in MSCs is regulated by mechanical stimuli induced by fluid shear stress in the bone marrow microenvironment.

### 2.4. Impact of Fluid Shear Stress on the Activity of HAS

**Figure 7:**
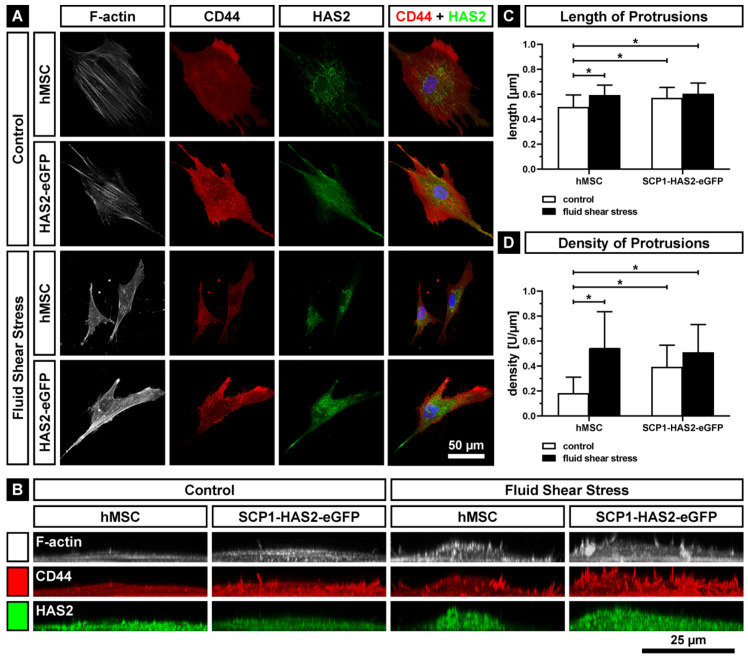
Formation of cell protrusions after the application of 20 h of fluid shear stress on primary hMSCs and HAS2-overexpressing immortalized hMSCs (SCP1-HAS2-eGFP). Cells were stained for confocal microscopy with phalloidin for F-actin (white); immunostained for CD44 (red) and HAS2 (green); and counterstained with DAPI for the nuclei (blue). (**A**) Z-stack maximum projection of fluid shear stress-stimulated and static (control) cells. (**B**) Y-stack maximum projection of shear stressed and control cells. Note the increased number of actin-containing membrane protrusions upon shear stress. (**C**) Length of protrusions. (**D**) Density of protrusions. Asterisks indicate a *p*-value < 0.05. Error bars represent SD.

In order to investigate whether the applied shear stress regulates the transport and the localization of the hyaluronan synthases in the plasma membrane, we performed immunofluorescence staining with antibodies against HAS2 and the HA receptor CD44 to delineate the cell membrane. In addition, actin cytoskeletal structures were visualized by phalloidin staining. As control, we used the previously described HAS2-overexpressing immortalized hMSC cell line, SCP1-HAS2-eGFP, in which the HAS2 gene is under the control of the constitutive cytomegalovirus promoter [62]. We observed that the application of fluid shear stress induced morphological changes in both primary hMSCs and SCP1-HAS2-eGFP cells. In general, top-view confocal microscopy images indicated a reduced cell area (Figure 7A), whilst side-view images revealed an increased height of the cells exposed to shear stress compared with static controls (Figure 7B). Due to these morphological changes upon the treatment with shear stress, we were not able to investigate the localization of HAS2 in detail. Indicating the overexpression of HAS2, the SCP1-HAS2-eGFP cells exhibited a stronger fluorescence signal for HAS2 compared to the primary cells, which exhibited a clearer pattern of distribution around the nucleus (blue-colored signal). The red CD44 staining showed bright spots corresponding to microvillus-like protrusions, visible as F-actin (white)-containing spikes on the cell surface in the side view of the cells (Figure 7B). The mean length of the protrusions in hMSCs (0.497 ± 0.098 µm) was significantly increased by the application of fluid shear stress (0.593 ± 0.079 µm), the overexpression of HAS2-eGFP (0.570 ± 0.084 µm) or a combination of both conditions (0.604 ± 0.086 µm) (Figure 7C). Under static conditions (control), the HAS2-eGFP-overexpressing cells displayed a significantly higher density of protrusions (0.392 ± 0.174 U/µm) compared to the primary hMSCs (0.182 ± 0.129 U/µm). Interestingly, fluid shear stress significantly increased the density of protrusions in hMSCs (0.545 ± 0.290 U/µm), but not in SCP1-HAS2-eGFP cells (0.510 ± 0.221 U/µm). A recent study by Koistinen et al. demonstrated that the formation of such protrusions in HAS3-overexpressing human breast adenocarcinoma cell line MCF-7 was triggered by active hyaluronan synthesis [63]. Since only HASes localized in the plasma membrane are active, our results also indicate that more HASes are transported to the plasma membrane because of the mechanical stimulation.

**Figure 8:**
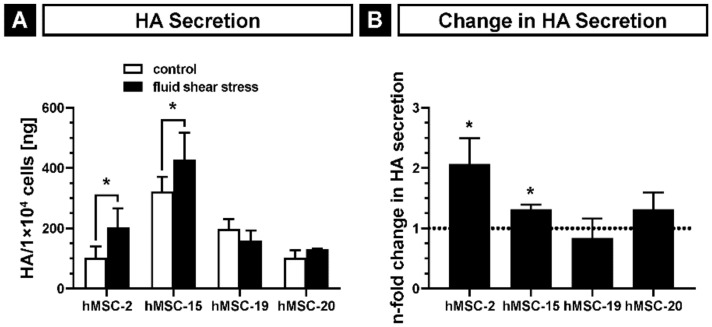
Hyaluronan synthase (HAS) activity in hMSCs cultured under fluid shear stress as determined by a hyaluronan ELISA assay-based quantification of secreted HA. (**A**) The graph shows the mean HA content per 1 × 10^4^ cells in the supernatant after 20 h of incubation in culture medium with or without the application of fluid shear stress; (**B**) n-fold change in the HA secretion of each donor due to shear stress. Asterisks indicate a *p*-value < 0.05. Error bars represent SD.

Furthermore, we determined the amount of secreted HA in the supernatants of fluid shear stress-treated hMSCs compared to the static cultured hMSCs using a commercial HA-ELISA kit. In the static cultures, the basic activity levels ranged between 101.6 ± 38.0 and 322.3 ± 48.8 ng HA/1×10^4^ cells among the donors. Consistent with this result, a donor-dependent variation of HAS activity has been previously reported [17]. Similarly, the HA levels in fluid flow-exposed hMSCs displayed a broad variance (130.5 ± 1.1 to 427.5 ± 89.4 ng HA/1×10^4^ cells) (Figure 8A). Nevertheless, focusing on the single donors, three out of the four donors (hMSC-2, hMSC-15 and hMSC-20) showed an upregulation of their HA secretion (1.32 ± 0.28- to 2.07 ± 0.43-fold), whilst one (hMSC-19) exhibited a modest downregulation (0.84 ± 0.32-fold change) (Figure 8B) after exposure to fluid shear stress. The upregulation of HA secretion was significant in hMSC-2 and hMSC-15 (*p* < 0.05).

Altogether, the increased number of microvillus-like protrusions and the slightly higher amount of secreted HA upon fluid shear stress indicate the mechanosensitivity of the HASes in hMSCs.

## 3. Discussion

Bone marrow-resident MSCs play an important role in bone formation and homeostasis. This function in vivo is mainly fulfilled by their osteogenic differentiation capacity through osteoprogenitor cells, pre-osteoblasts and mature osteoblasts. It has been previously reported that MSC differentiation into the osteogenic lineage in vitro can be induced by the application of different types of mechanical stimulations such as matrix strains and shear stress [64]. In vivo, bone cells and hMSCs are exposed to fluid shear stress due to the lacunae and canaliculi that serve as a channel system for fluids in the bone [65]. Fluid flow is generated by changes in blood pressure due to muscle contractions and due to mechanical loading and can reach pressure levels over 10 Pa in the bone marrow of mammals [10,66]. In hMSCs, the primary cilium is suspected to be the main sensor for the mechanoperception of fluid shear stress [37]. In the present study, we demonstrated that our experimental system drives a cellular and molecular response of hMSCs upon mechanical stimulation induced by steady fluid flow. We observed a clear morphological change from a cuboidal geometry to a more hydrodynamic, spindle-like shape. The changes in the gene expression level were proven by the enrichment of different gene sets linked to mechanoperception, such as the cellular response to fluid shear stress (GO:0071498), or by the upregulation of the expression of *PTGS2*, which is a well-known marker for the application of this stimulus in hMSCs [37]. Beside the direct response to the fluid flow-induced mechanical signals, gene sets that are linked indirectly to fluid shear stress also show an enrichment, particularly reactions to changed physical conditions via cell cycle arrest (GO:0007050), downregulation of motility (GO:2000146), upregulation of adhesion (GO:0045785) and changes in intercellular adhesions (GO:0022407).

Commitment towards the osteogenic lineage in hMSCs can be achieved via mechanotransduction [53], applying various types of physical stimuli such as tension [67], compression [68,69] and fluid shear stress [70]. Although a variety of mechanosensors have been described for different bone cells, e.g., integrins and cadherins/β-catenin in osteoblasts [71,72,73,74], the aforementioned mechanoperception by the primary cilium is the key player in hMSCs [37,75], resulting in an extensive upregulation of the expression levels of *PTGS2* and *BMP2*. Signal transduction of mechanically stimulated osteogenesis in hMSCs is mediated by MAP kinase pathways [76] and Wnt signaling [31,32]. *PTGS2*, *BMP2*, *WNT1* and *WNT16* are part of the 25 most upregulated genes in our dataset, whilst *BMP6*, *WNT10B* and *BMPR1B* were at least part of the 624 significantly upregulated genes as well as part of gene sets linked to osteogenesis. According to the literature, we observed enrichments in the cellular response to a BMP stimulus (GO:0071773) without the addition of these proteins, the upregulations of osteoblast differentiation (GO:0045669), biomineral tissue development (GO:0070169), bone mineralization (GO:0030501) and ossification (GO:0045778) and skeletal system morphogenesis (GO:0048705).

In bone marrow-derived hMSCs, all three HAS isoenzymes (HAS1, HAS2 and HAS3) are expressed, while HAS2 is the most abundant isoform and HAS3 has the lowest expression level [17,62]. The present study confirms these results by determination of the transcript expression levels of *HASes* in hMSCs obtained from different donors (see Supplementary Materials). We have also found that the differential expression levels of *HAS1* and *HAS2* were significantly upregulated in hMSCs upon shear stress. The lowest expressed isoform *HAS3* showed a positive tendency in only one donor. Previously, it was demonstrated that the expression of HAS2 is induced by pulsatile, arterial-like shear stress, but not by lower shear stress in human umbilical vein endothelial cells (hUVECs) [52]. To our knowledge, we confirmed, for the first time, that the expression of HAS1 and HAS2 is influenced by shear stress in hMSCs. In endothelial cells, HAS expression and HA production are regulated by KLF2-mediated glycolysis [77]. In our study, *KLF2* is significantly upregulated (Figure 4B), but we also observed an upregulation of cytokine mRNAs regulating HAS expression such as *PDGF-BB* and *IL1-β* (Figure 2D).

Our data strongly suggest that fluid shear stress not only upregulates the mRNA expression of HASes but it also increases their activities. On the one hand, the HA content in the supernatants of hMSCs cultured in the presence of shear stress was higher than in the supernatants of hMSCs cultured under static conditions (Figure 8). On the other hand, we observed the formation of microvillus-like spikes on the surface of the cells treated with shear stress (Figure 7). At first glance, these structures do not have any physical meaning since they are formed against the shear stress flow without adherence to the substratum. However, such structures are known to be induced by increased activity of HASes [63]. HAS2-overexpressing hMSCs, which have an increased HAS activity in comparison to non-transduced hMSCs [62], display a higher number of protrusions with and without shear stress. This indicates that the formation of the protrusions depends on the activity of HASes in our study. Since we were not able to build up the flow system under the confocal microscope, we could not monitor the microvilli-like structures in alive hMSCs. Although the fixation procedure disturbs the length and the density of the plasma membrane protrusions, they can be used for a comparative analysis as they still show the relative differences between the analyzed groups [63,78,79]. The length and density of the protrusions might also depend on the cell type. Our data suggest a reduced actin depolymerization due to the upregulation of *XIRP1*, an inhibitor of actin depolymerization. The consequence might be the increased attachment of the shear stress-induced cells as indicated by the negative regulation of cell motility (GO:2000146) and the positive regulation of cell adhesion (GO:0045785). However, there could also be a correlation with the increased formation of HAS-induced protrusions which are known to contain actin filaments in themselves and in their cortical bases [63]. We also observed actin filaments mainly in the bases of the protrusions (Figure 7).

The molluscan myosin-chitin synthases are assumed to be involved in the formation of actin-rich microvilli at the shell-forming interface [80]. As the chitin synthases might be localized in the tips of these microvilli, HASes and myosin 10 are located in the protrusions of HA-producing cells [63]. These arrangements of the glycosyltransferases could have consequences for transducing shear and adhesive forces between the mineralizing composite and the material forming cells [80].

The exact function of HA in hMSCs is not fully understood yet. HA might regulate their adhesive properties in the bone microenvironment [62], but it could also play a crucial role in the mineralization process of the bone. In this work, we demonstrated that the expression and activity of HASes are regulated by fluid shear stress in bone marrow-derived hMSCs, concomitant with the upregulated expression levels of genes related to their osteogenic potential. Taking into account that genetically diverse MSC subpopulations in the bone marrow likely experience various types of fluid shear stress with different magnitudes and durations, our study has an obvious limitation when interpreting the in vitro experimental data for the in vivo situation. Therefore, further studies are needed to elucidate the relevance of HA in the bone microenvironment.

## 4. Materials and Methods

### 4.1. Cell Isolation and Culture

Human MSCs were isolated from the bone marrow of femoral heads of four healthy male donors (49 to 87 years old) recruited at the Clinic for General, Trauma and Reconstructive Surgery of the Ludwig-Maximilians-University (LMU, Munich, Germany). The study was approved by the ethics committee of Ludwig-Maximilians-University, Munich (project code 19-177, 24 July 2019) and performed according to the Declaration of Helsinki. A written informed consent declaring that the eliminated tissue can be used for medical studies at the university hospital was signed by all donors. The cell isolation was performed by washing the bone graft material with DPBS (pH 7.4, Biochrom, Berlin, Germany). Afterwards, the bone material was incubated with 250 U/mL collagenase II (Worthington Biochemical Corp., Lakewood, NJ, USA) in DMEM (Life Technologies, Carlsbad, CA, USA) three times for 10 min each at 37 °C. Both suspensions were filtered with a 100-μm cell strainer to remove bone fragments. After centrifugation at 500× *g* for 5 min, the cell pellet was resuspended in a culture medium consisting of MEM α with nucleosides and GlutaMAX supplement (Life Technologies) containing 10% (*v*/*v*) fetal bovine serum (Sigma-Aldrich, St. Louis, MO, USA), and 1000 U/mL penicillin and 1000 µg/mL streptomycin (Biochrom) at 37 °C with 5% CO_2_ and ~90% humidity. Nonadherent cells were removed by washing with DPBS for three times after the first three days in culture. Cells were passaged when reaching a confluence of ~80% and frozen at passage three in culture medium containing additional 20% (*v*/*v*) FBS and 10% (*v*/*v*) dimethyl sulfoxide (Applichem, Darmstadt, Germany) in liquid nitrogen. Aliquots were thawed and cultured five days in advance to the experiments, which were always performed with cells in passage four.

The generation and culture conditions of the HAS2-overexpressing immortalized hMSCs (SCP1-HAS2-eGFP) are described elsewhere [62].

### 4.2. Application of Fluid Shear Stress

To apply fluid shear stress as a stimulus of mechanotransduction in hMSCs, a setup for culture under flow conditions was created (Figure 1A). Therefore, a µ-Slide I 0.2 Luer ibiTreat (Ibidi, Planegg, Germany) was connected via a silicone tubing with 1.6 mm inner diameter (Ibidi) with a medium reservoir in a Nalgene cell culture medium bottle (Thermo Fisher Scientific, Waltham, MA, USA) containing 50 mL culture medium with two connectors and a pressure equalization set (0.22 µm sterile filter) on the GL45 lid (DWK Life Sciences, Wertheim, Germany) for medium circulation. Downstream of the reservoir and upstream of the channel slide, a PharMed Ismaprene pump tubing with 2.79 mm inner diameter (Ismatec, Wertheim, Germany) was inserted, to connect an IPC-8 peristaltic pump (Ismatec) to drive the medium flow. The pump was calibrated to create a fluid shear stress of 10 Pa (100 dyn/cm^2^) inside the channel slide according to the manufacturer’s instructions to reach the level of volumetric mean marrow shear stress in porcine femurs [10]. All components beside the pump were kept under the cell culture conditions in the incubator and put there, altogether with the medium, 24 h in advance to the experiment. Beside the channel slides, 24-well plates (Nunc, Darmstadt, Germany) or 8-well chamber slide ibidiTreat (Ibidi) was used for the static control cells, which were all coated with 2 µg/cm^2^ human plasma fibronectin (Millipore, Billerica, MA, USA) in DPBS for 1.5 h at 37 °C and washed with DPBS. The number of seeded cells was: 2.5×10^5^ cells per channel slide; 1×10^5^ cells per well of the plate; 5×10^4^ cells per well of the chamber. After 6.5 h of adhesion, the medium was changed in the control wells and the channel slides were connected to the flow setup to apply shear stress for 20 h. Before and after the mechanical stimulation, images were taken from both conditions using an Axiovert 40 CFL microscope (Carl Zeiss, Oberkochen, Germany).

### 4.3. Next-Generation RNA Sequencing and Bioinformatics

The cells from all four donors were lysed in 200 µl Trizol (Life Technologies) per well (24-well plate)/channel slide. After RNA isolation by following a standardized protocol, RNA quality and quantity were measured using a BioAnalyzer (Agilent, Santa Clara, CA, USA). The libraries for sequencing were prepared with a SENSE mRNA-Seq Library Prep Kit V2 (Lexogen, Vienna, Austria) and sequenced on a HiSeq1500 device (Illumina, San Diego, CA, USA) with a read length of 50 bp and a sequencing depth of approximately 15 million reads per sample. The raw basecall (Bcl) files were demultiplexed with Illumina_Demultiplex. Transcriptomes were aligned to the human reference genome GRCh38.99 by using STAR (version 2.7.2b) [81] to obtain the reads per gene.

DESeq2 (version 1.26.0) [82] was used to analyze the results of RNA sequencing and the comparison of differentially expressed genes in between treatment methods run on R version 3.6.1. Low gene expressions were filtered through elimination of row counts by a cutoff of a minimum 10 reads per gene, which resulted in the remaining 21,451 genes. The data were normalized through variance-stabilizing transformation (vst), and for a principal component analysis (PCA), the vst data were transformed to log_2_. Differentially expressed genes were obtained through DESeq2 and insignificant genes were filtered by a 0.05 adjusted *p*-value cutoff. Log_2_-fold changes (LFC) smaller than -2 or higher than 2 were defined as thresholds to identify biologically relevant results. From 21,451 genes, 624 were considered to be upregulated and 683 were downregulated after application of the fluid stress (Figure 2B).

Up- and downregulated genes in the DESeq2 results were analyzed for their Biological Process Gene Ontology Results (GO) by using clusterProfiler (v3.14.3) [83] with org.Hs.eg.db_3.10. The datasets of pathways of interest are involved in the skeletal system, biomineralization, HA biosynthesis and, to indicate the reaction to the mechanical stimulus, the response to fluid shear stress. The enrichment scores were calculated by dividing the sample frequency by the background frequency of genes annotated to a certain GO term. The sample frequency is the number of genes annotated to a certain GO term in the input list divided by the number of all genes in the input list (adjusted *p* ≤ 0.05; log_2_-fold change ≥2; part of the background set provided by clusterProfiler). The background frequency is the number of all genes annotated to a GO term divided by the number of genes of the entire background set provided by clusterProfiler.

The expression of HA-related genes, a subset consisting of the hyaluronan biosynthetic process (GO:0030213) and five HA receptors (*CD44*, *HMMR*, *ICAM1*, *LAYN* and *LYVE1*) obtained from the DESeq2 results was analyzed concerning the LFC and adjusted *p*-values. In a similar manner, the change in expression levels in the subset of genes corresponding to the cellular response to fluid shear stress (GO:0071498) was used to verify mechanotransduction.

### 4.4. Immunostaining, Confocal Microscopy and Quantification of Protrusions

Fluorescence staining of primary hMSCs and SCP1-HAS3-eGFP cells was performed to analyze the morphological changes at the molecular level after the application of fluid shear stress. Therefore, 1×10^3^ cells were seeded in 300 µl culture medium per well of a fibronectin-coated 8-well chamber slide as static control, and 2.5×10^5^ or 1.25×10^4^ cells on two fibronectin-coated channel slides. The complete experimental procedure was performed as described above: the two µ-slides were connected in a row, with the higher cell density upstream. The staining was performed on the slide with the lower cell density. After 20 h in the experimental setup, the cells were washed with DPBS and fixed for 10 min at room temperature with 4% (*w/v*) paraformaldehyde in DPBS, followed by a permeabilization step with 0.1% (*v/v*) Triton X100 (Sigma-Aldrich) in DPBS per well/channel. After an additional washing step with DPBS, the cells were blocked with 1% (*w*/*v*) BSA (Sigma-Aldrich) in DPBS for 1 h at room temperature. Following this, a rabbit anti-HAS2 antibody (1:100, orb157430, Biorbyt, Cambridge, UK) and a mouse anti-CD44 antibody (1:400, #3570, Cell Signaling Technology, Cambridge, UK) were diluted in 1% BSA in DPBS. The fixed cells were incubated over night at 4 °C in primary solution. Afterwards, the wells were washed with 1% BSA in DPBS. As secondary antibodies, a goat anti-rabbit Alexa Fluor 488 conjugate (4 µg/mL, A10036, Thermo Fisher Scientific), a donkey anti-mouse Alexa Fluor 546 conjugate (4 µg/mL, A11008, Thermo Fisher Scientific) and a phalloidin Alexa Fluor 647 conjugate (1:50, A22287, Thermo Fisher Scientific) diluted in 1% BSA in DPBS were added for 1 h at room temperature. The cells were washed with DPBS before the nuclei were counterstained for 2 min with 4,6-diamidino-2-phenylindole (DAPI) and washed a final time with DPBS. Finally, the stained cells were kept in DPBS to perform confocal microscopy using a Leica SP8 AOBS WLL, a HC PL APO 63×/1.30 GLYC CORR CS2 objective and Lightning deconvolution software applying 1.28× zoom (all from Leica Microsystems, Wetzlar, Germany). Quantification of protrusions and the definition of density (ratio of detected protrusions to cell edge length) were performed as described elsewhere [78] using the FiloQuant plugin [84] for the FIJI software [85]. Therefore, the single image analysis tool was used in combination with manual outlining of the cell edges on y-stack maximum projection images based on the CD44 staining. For quantification, 30 to 48 cells per group (hMSC-2, hMSC-15 and SCP2-HAS2-eGFP) were analyzed. The statistical analysis was performed using the GraphPad Prism software (GraphPad Software, San Diego, CA, USA) to perform a Kruskall–Wallis test with Dunn’s multiple comparison test. A *p*-value less than 0.05 was considered as statistically significant.

### 4.5. HA-ELISA

HAS activity was analyzed by measuring the HA content in the supernatant of the cells cultured with fluid shear stress and cells cultured in 8-well slides as static control. As substrate, 10 mM N-acetyl-D-glucosamine (Sigma-Aldrich) was added to the standard culture medium for 20 h during the experiment. Afterwards, the supernatant was collected. As described above, the cells were washed, fixed and stained with a phalloidin Alexa Fluor 488 conjugate (1:400, A12379, Thermo Fisher Scientific) diluted in DPBS for 1 h at room temperature. After washing in DPBS, the nuclei were counterstained with DAPI. Finally, mosaic pictures of the whole growth area were taken using an AxioObserver Z1 (Carl Zeiss). The cells were counted using the FIJI software [85]. The quantification of the HA content in the supernatant was performed using the commercial enzyme-linked immunosorbent assay (ELISA) TECO Human Hyaluronic Acid Test TE1017-2 Kit (TECOmedical, Bünde, Germany) according to the manufacturer’s instructions. The absorbance was determined with a photometric plate reader MultiscanFC (Thermo Fisher Scientific). Two wells per experiment were measured, and the whole experiment was performed three times per donor. The statistical analysis was performed using the GraphPad Prism software (GraphPad Software, San Diego, CA, USA) to test for Gaussian distribution and to perform a paired *t*-test. A *p*-value less than 0.05 was considered as statistically significant.

## Data Availability

All raw sequencing data are available upon request and approval of the local ethics committee.

## Supplementary Materials

A list with the LFC, *padj* and transcripts per million for all above mentioned genes and a figure showing the individual changes of vst values can be found at https://www.mdpi.com/1422-0067/22/6/3123/s1.

## Abbreviations

BMP	Bone morphogenetic protein
CMV	cytomegalovirus
HA	hyaluronan
HAS	hyaluronan synthases
hMSCs	human mesenchymal stem cells
hUVECs	human umbilical vein endothelial cells
LFC	log_2_-fold change
min	minutes
*Padj*	adjusted *p*-value
RNAseq	RNA sequencing
SD	standard deviation
SE	standard error
